# Redefining Clinical Perspectives on MCS: Toward an Evidence-Based, Multisystem Model. Comment on Jacques, L. Multiple Chemical Sensitivity: A Clinical Perspective. *Brain Sci.* 2024, *14*, 1261

**DOI:** 10.3390/brainsci15070747

**Published:** 2025-07-14

**Authors:** Elaine Psaradellis

**Affiliations:** Environmental Health Association of Québec, P.O. Box 364, Saint-Sauveur, QC J0R 1R0, Canada; elaine@aseq-ehaq.ca

The article “Multiple Chemical Sensitivity: A Clinical Perspective”, published in *Brain Sciences*, suggests that multiple chemical sensitivity (MCS) is primarily a psychogenic disorder, rooted in unresolved emotional trauma and stress responses [1]. However, emerging scientific evidence points to a more complex, multifactorial understanding of MCS that challenges this model. While psychological factors, such as stigma and the emotional toll of managing a chronic condition, undoubtedly influence the experience of MCS, it is critical to recognize that MCS cannot be reduced to a psychological phenomenon. Increasingly, MCS is being recognized as a condition shaped by environmental, biological, and physiological mechanisms. Notably, this broader understanding is reflected in international frameworks, including the recent recognition of MCS-related access barriers in the Concluding Observations by the UN Committee on the Rights of Persons with Disabilities [2].

One of the central arguments in the article is that MCS is a psychogenic disorder, emphasizing the role of psychological factors in its onset and manifestation. However, this emphasis overlooks a substantial body of evidence supporting the physiological basis of MCS. Recent research synthesize findings from a substantial body of experimental and observational studies reporting objective physiological markers, such as elevated oxidative stress, altered neural activation, and receptor sensitization, particularly involving TRPV1 and TRPA1 receptors [3]. These findings suggest that MCS involves complex physiological interactions. Notably, receptor sensitization has been identified as a key physiological mechanism in MCS. In fact, 21 studies have highlighted receptor sensitization as a significant factor in MCS pathophysiology, further reinforcing the idea that MCS has a strong biological foundation [3]. Recent research has demonstrated that improving indoor air quality (IAQ) by reducing volatile organic compounds (VOCs) led to significant symptom improvement in individuals with chemical intolerance, further reinforcing the biological foundation of MCS [4]. This is an important area of research that continues to evolve, with implications for both clinical diagnosis and treatment.

The legal landscape increasingly acknowledges the physiological basis of MCS, including mechanisms like receptor sensitization. Three significant workplace accommodation cases have established strong legal precedents, drawing on compelling evidence of chemical exposure and biological responses. These rulings, supported by case documents [5,6,7], reflect the growing recognition of MCS as a medical condition rooted in physiological processes.

The article also fails to adequately consider the substantial body of research linking MCS to environmental exposures and genetic predispositions. Case–control and population-based studies have shown that certain gene variants may increase susceptibility to chemical intolerance. For example, McKeown-Eyssen et al. found significant associations between MCS and genetic polymorphisms in xenobiotic metabolism genes such as CYP2D6 and NAT2, with carriers of active CYP2D6 and rapid NAT2 variants showing 3- to 4-fold increased odds of MCS, highlighting a likely heritable component to chemical sensitivity [8]. In a large survey of over 10,000 U.S. adults, Miller et al. found that individuals with higher mast cell activation syndrome (MCAS) scores were significantly more likely to meet criteria for chemical intolerance, with those in the highest MCAS quartile having over six times greater odds of chemical intolerance compared to the lowest quartile, underscoring the strong link between immune activation and chemical sensitivity at the population level [9]. Palmer et al. further identified genetic factors associated with chemical intolerance, including genes linked to mast cell signaling, and showed that exposures such as bisphenol A and benzo(a)pyrene may modulate gene expression, supporting a gene–environment interaction model for MCS [10]. Collectively, these studies provide consistent evidence across multiple populations linking genetic factors and environmental exposures to chemical intolerance. However, further research is needed to validate and clarify these complex interactions.

Building on this growing body of evidence, it is important to note that countries such as Germany, Japan, and Spain have officially recognized MCS as an environmental health condition, incorporating it into their medical classifications with ICD-10 codes. This recognition highlights the need for a more comprehensive understanding of MCS, one that integrates both environmental triggers and genetic susceptibility.

Furthermore, the article’s inclusion of three case studies, which are purported to represent MCS, warrants further examination. Two of these cases appear to describe symptoms that result from carbon monoxide (CO) poisoning—an entirely distinct condition from MCS, as defined in the 1999 MCS Consensus Definition. This definition was selected as it has demonstrated strong discriminative validity, effectively distinguishing MCS from other conditions in clinical populations [11]. At the same time, the absence of detail regarding case selection and documentation procedures makes it difficult to assess the evidentiary strength of these examples. Clarifying how and why these particular cases were chosen would help determine whether they accurately reflect the broader clinical picture of MCS or if they risk introducing unintended bias.

In light of the above, we call for a broader more evidence-based approach to the study of MCS that does not narrowly focus on psychogenic explanations. We recommend that research and publications incorporate both the environmental and physiological dimensions of MCS while also acknowledging the psychological effects that result from the stigmatization of individuals with the condition. It is crucial that the scientific community engages with a more comprehensive framework for understanding MCS which includes both empirical studies and the perspectives of those living with the condition.
